# Comparative Study of Phosgene Gas Sensing Using Carbon and Boron Nitride Nanomaterials—A DFT Approach

**DOI:** 10.3390/molecules26010120

**Published:** 2020-12-29

**Authors:** Emmanuel Obroni Kweitsu, Stephen Kanga Armoo, Kwabena Kan-Dapaah, Eric Kwabena Kyeh Abavare, David Dodoo-Arhin, Abu Yaya

**Affiliations:** 1 Department of Materials Science and Engineering, School of Engineering Sciences, CBAS, University of Ghana, Legon P.O. Box LG 77, Ghana; ekweitsu@gmail.com (E.O.K.); ddodoo-arhin@ug.edu.gh (D.D.-A.); 2 Department of Computer Engineering, School of Engineering Sciences, CBAS, University of Ghana, Legon P.O. Box LG 77, Ghana; SArmoo@ug.edu.gh; 3 Department of Biomedical Engineering, School of Engineering Sciences, CBAS, University of Ghana, Legon P.O. Box LG 77, Ghana; KKan-Dapaah@ug.edu.gh; 4 Department of Physics, Ghana Private Mail Bag, Kwame Nkrumah University of Science and Technology, Kumasi 00233, Ghana; EKKAbavare.cos@knust.edu.gh

**Keywords:** phosgene, boron nitride, carbon nanotube, DFT, LDA

## Abstract

Phosgene (COCl_2_), a valuable industrial compound, maybe a public safety and health risk due to potential abuse and possible accidental spillage. Conventional techniques suffer from issues related to procedural complexity and sensitivity. Therefore, there is a need for the development of simple and highly sensitive techniques that overcome these challenges. Recent advances in nanomaterials science offer the opportunity for the development of such techniques by exploiting the unique properties of these nanostructures. In this study, we investigated the potential of six types of nanomaterials: three carbon-based ([5,0] CNT, C60, C70) and three boron nitride-based (BNNT, BN60, BN70) for the detection of COCl_2_. The local density approximation (LDA) approach of the density functional theory (DFT) was used to estimate the adsorption characteristics and conductivities of these materials. The results show that the COCl_2_ molecule adsorbed spontaneously on the Fullerene or nanocages and endothermically on the pristine zigzag nanotubes. Using the magnitude of the bandgap modulation, the order of suitability of the different nanomaterials was established as follows: PBN60 (0.19%) < PC70 (1.39%) < PC60 (1.77%) < PBNNT (27.64%) < PCNT (65.29%) < PBN70 (134.12%). Since the desired criterion for the design of an electronic device is increased conductivity after adsorption due to the resulting low power consumption, PC60 was found to be most suitable because of its power consumption as it had the largest decrease of 1.77% of the bandgap.

## 1. Introduction

Phosgene, is a valuable industrial organic compound, with a chemical formula COCl_2._ It was first obtained in 1812 by John Humphrey Davy when he exposed a mixture of chlorine and carbon monoxide to sunlight [1]. At room temperature, it appears as a colourless gas and at low concentrations, smells like newly cut musty hay. It is a highly toxic substance that can lead to pathophysiological conditions such as blurred vision, irritable eyes, throat, and lungs, asphyxia, and even death when inhaled at low concentrations [2]. These harmful properties of COCl_2_ can, and in fact, has been exploited as a chemical weapon agent. One notable instance is when the Germans used it as a choking agent during World War I in 1915 causing about 80% of all chemical-related casualties at the time [3]. Today, the threat of this insidious property can be related to potential industrial accidents or leakages as well as terrorist attacks on industrial plants, stocks, or transports. Therefore, there is a need for the development of detection techniques for public safety in an industrial environment or against terrorist attacks. Conventional methods based on techniques—such as molecular spectrometry, electrochemistry, and mass spectrometry combined with chromatography [4,5,6,7,8,9,10]—suffer issues related to procedural complexity and sensitivity.

Recent advances in nanomaterials science offer the opportunity for the design of simple, smart, and sensitive gas sensors due to their easy preparation methods and unique properties to address these challenges. The choice of nanomaterials is based on the historical performance in adsorption studies related to their potential to detect gas molecules in general and phosgene gas in particular [11,12,13,14,15,16]. Different kinds of nanomaterials have been extensively used to detect toxic and pollutant gases [17]. The gas sensing mechanism of nanomaterials is based on their surface reactivity, which is highly influenced by factors such as surface capping, structure-directing agents, modifying morphology, catalysis, and working temperature [12]. Doped nanomaterials have been shown to have better gas sensitivities than pristine nanomaterials at optimum working temperature. Several studies reported in the literature on finding prospective nanomaterials for phosgene gas sensing have sought to exploit the modulation in the conductivity of the phosgene-nanomaterial adsorption system. The desired characteristic of the nanomaterial is the modulation of the electrical properties of the phosgene-adsorbent system resulting in a reduction of the bandgap and an increase in the adoption energy [11,16].

The search for candidates for the adsorption of phosgene gas also considered BN24 and BN32 and single-walled boron nitride nanotubes [11,18,19] for their suitability. DFT simulations indicate that BN nanotube band gap is independent of the tube diameter, chirality and whether the nanotube is single-walled, multiwalled, or packed in bundles. Boron nitride nanotubes are always semiconducting with a large bandgap of about 5.5 eV [20,21]. The nanotubes also showed a considerable electronic response and proved suitable for sensing applications of CO_2_ and ethyl benzene gases [19,22]. These studies were conducted using higher-levels of functionals of the density functional theory. The higher- level functionals were preferred by all the researchers under review because of reported higher accuracy. These functionals, though more accurate, have higher computational cost. Additionally, BN60 and BN70 have not been studied for suitability of phosgene gas sensing. Carbon nanotubes especially for those of the zigzag type, C60 and C70 have also shown some promise as having the electrical properties that allow for sufficient modulations to their band structure after adsorption of phosgene gas [18,19,20,21,22,23,24]. Carbon nanotubes can be either semiconducting or metallic, depending on the chirality of the tube [25]. A recent study conducted by Elloh et al. suggested that C60 had significant bandgap tuneable properties when PVK/C60 was studied under the DFT/LDA and generalized gradient approximation framework using Perdew, Burke, and Ernzerhof (GGA-PBE) [24]. Whiles the literature points to the potential of these nanomaterials as suitable, a comparative study of these materials in their pristine form for sensing phosgene is yet to be studied, hence the significance of this investigation.

## 2. Computational Methods

Computations under DFT/LDA approach were done using the Quantum Espresso Software based on Kohn Sham formulation [26,27,28]. All the electron calculations were carried out using Plane-Wave in combination with Ultrasoft Pseudopotentials [29]. The models were constructed using the QuantumATK Atomistic Simulation Software. For each model, the geometry was optimized and K- Points and kinetic energy-cut convergence test were done to obtain accurate ground state energy. The atomic positions and cell parameters were relaxed in all cases. Next, we sampled with Γ-centred high-symmetry paths of Γ−X for pristine [5,0] CNT, [5,0] BNNT nanotubes and Γ−M−K−L−Γ for phosgene-[5,0] CNT and phosgene-[5,0] BNNT cluster and Γ−R−T−U−V−X−Y−Z [30] for pristine and phosgene [C60, C70, BN60] clusters in order to generate the band structures. The Brillouin zone was sampled under the Monkhorst pack grid scheme. Visualizations were done using the Xcrysden software and computational resources was provided by the Lengau High-Performance Computing Cluster, South Africa.

### Adsorption Model

The adsorption model is described in the equation below as follows.

(1) $$E_{ads}=E_{Phosgene_{-adsorbent}}-\left(E_{phosgene}+E_{adsorbent}\right)$$

where *E_ads_* is the adsorption energy, *E_phosgene-adsorbent_* is the cluster energy, and *E_Phosgene_* is the ground state total energy of the relaxed phosgene molecule and *E_adsorbent_* is the energy of the relaxed adsorbent molecule under study.

## 3. Results and Discussion

The results presented subsequently indicate except for [5,0] boron nitride nanotube and [5,0] carbon nanotube that the fullerene C60, C70, and the fullerene-like BN60 and BN70 may be suitable candidates for the sensing of phosgene gas. Though there is a substantial modulation in the bandgap of the nanotubes, the adsorption energies calculated showed that the energy barrier is potentially too high. In contrast, there was free energy available for the fullerene structures. Indeed, the magnitude of the changes in the band gaps was small but maybe found appreciable.

### 3.1. Phosgene on [5,0] CNT

**Figure 1:**
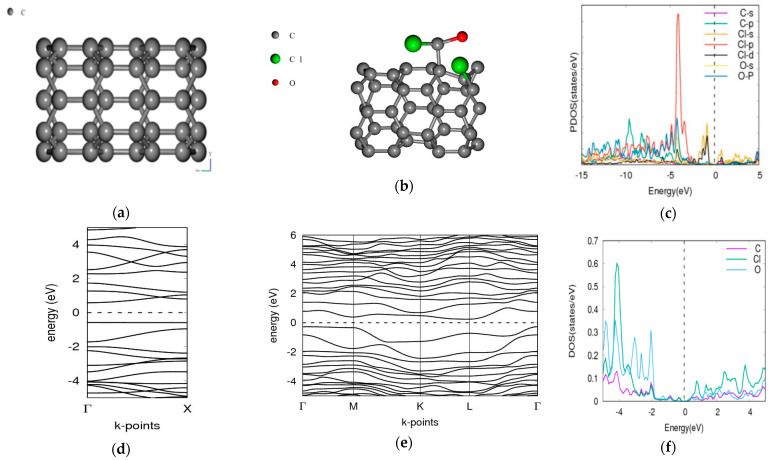
Band structure and density of states for phosgene gas interaction with [5,0] CNT. (**a**) CNT structure Eg = 1.8959 eV, (**b**) phosgene-[5,0]-CNT Eg = 3.1337 eV, (**c**) phosgene-CNT PDOS, (**d**) band structure of CNT, (**e**) band structure of phosgene-CNT, and (**f**) phosgene-CNT DOS.

The bandgap of the pristine [5,0] CNT is 1.8959 eV. This falls within a reasonable range when compared with 2.03/2.32 eV for tight binding calculations and DFT [31]. When the phosgene was adsorbed as shown in Figure 1, the combined band gap increased to 3.1337 eV indicating an increase of (65.29%). The energy difference between the composite CNT and phosgene was found to be 142.09 Ry.

### 3.2. Phosgene on [5,0] BNNT

**Figure 2:**
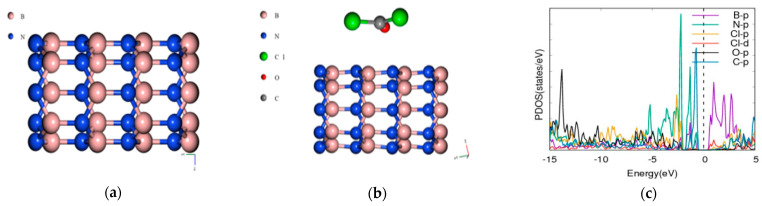

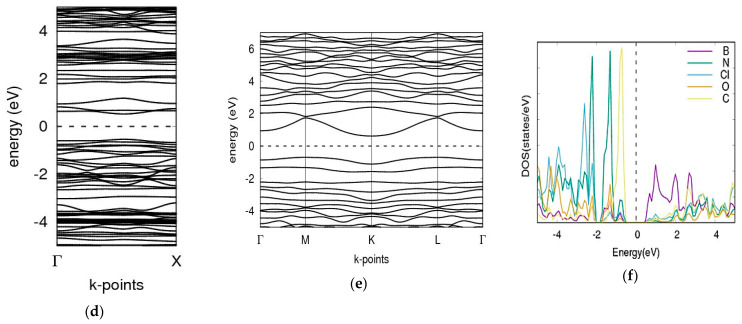
Band structure and density of states for BNNT and phosgene gas interaction. (**a**) [5,0] Boron nitride nanotube (BNNT), (**b**) phosgene-BNNT, (**c**) phosgene-[5,0] BNNT PDOS, (**d**) BNNT band structure Eg = 3.4181 eV, (**e**) phosgene-BNNT Eg = 4.3627 eV, (**f**) phosgene-BNNT DOS.

The bandgap of the pristine BNNT is 3.4181 eV when the phosgene was adsorbed as indicated in Figure 2, the combined band gap increased to 4.3627 eV indicating an increase of 27.64%. The energy difference between the composite of BNNT and phosgene was found to be 136.96 Ry.

### 3.3. Phosgene on C70

**Figure 3:**
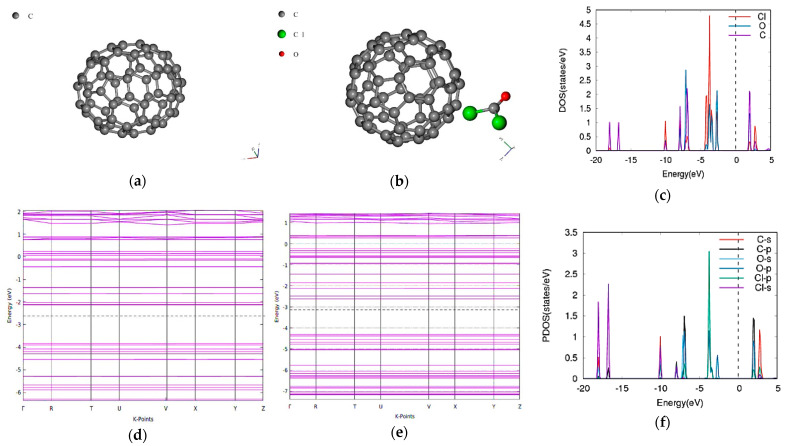
Band structure and density of states for phosgene gas interaction with C70. (**a**) C70, (**b**) phosgene-C70, (**c**) phosgene-C70 DOS, (**d**) C70 band structure Eg = 1.7273 eV, (**e**) phosgene-C70 Eg = 1.7033 eV, (**f**) phosgene-C70 PDOS.

The bandgap of the pristine C70 is 1.7273 eV consistent with other calculations both experimental [32] and computationally simulated [33]. When the phosgene was adsorbed, the combined bandgap decreased to 1.7033 eV as shown in Figure 3 indicating a decrease of −1.77%. The energy difference between the composite of C70 and phosgene was found to be −1.93 Ry.

### 3.4. Phosgene on C60

**Figure 4:**
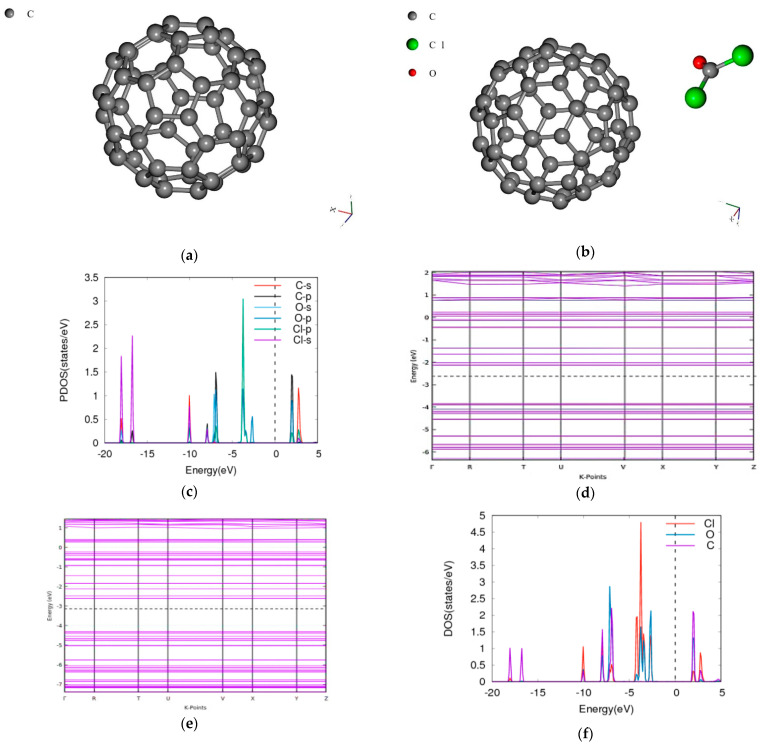
Band structure and density of states for phosgene gas interaction with C60. (**a**) C60, (**b**) phosgene- C60, (**c**) phosgene-C60 PDOS band-gap is (**d**) C60 band structure Eg = 1.6358 eV, (**e**) phosgene-C60 Eg = 1.6069 eV, (**f**) phosgene-C60 DOS.

The bandgap of the pristine C60 is 1.6358 eV and it agrees well with the experiment using the microwave absorption method [32]. Other computationally determined results reported it as 1.83 eV [34] using Minnesota 2006 local functional (M06-L) local meta-generalized gradient approximation (meta-GGA) functional and consistent with experimental value of 1.80 eV obtained through electron-energy-loss spectroscopy (EELS) [35]. When the phosgene was adsorbed, the combined bandgap decreased to 1.6069 eV as shown in Figure 4 indicating a decrease of −1.77%. The energy difference between the composite of C60 and phosgene was found to be −1.9332 Ry.

### 3.5. Phosgene on BN60

**Figure 5:**
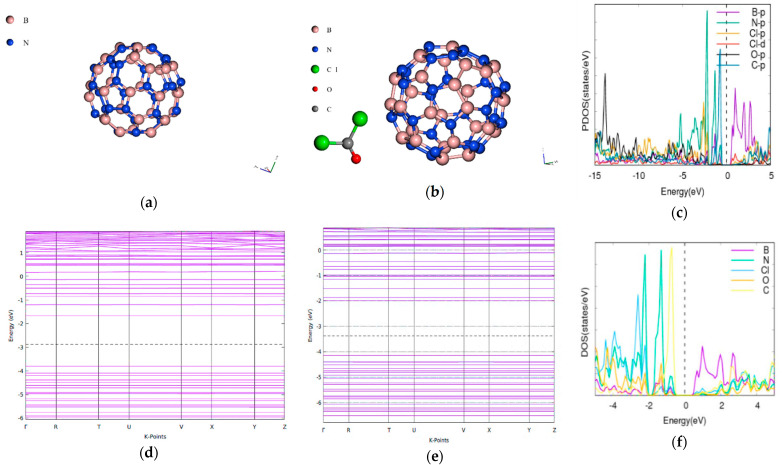
Band structure and density of states for phosgene gas interaction with BN60. (**a**) Pristine BN60, (**b**) phosgene-BN60, (**c**) phosgene-BN60 PDOS band-gap is (**d**) BN60 band structure Eg = 2.1454 eV, (**e**) phosgene-BN60 Eg = 2.1495 eV, (**f**) Phosgene-BN60 DOS.

The bandgap of the pristine BN60 is 2.14539 eV a smaller value compared with 2.55 eV obtained using the VASP implementation of DFT with valence electrons described by projector augmented wave (PAW) [36]. When the phosgene was adsorbed, the combined band gap increased to 2.1495 eV as indicated in Figure 5. This is an increase of 0.19%. The energy difference between the composite of BN60 and phosgene was found to be −1.9396 Ry.

### 3.6. Phosgene on BN70

**Figure 6:**
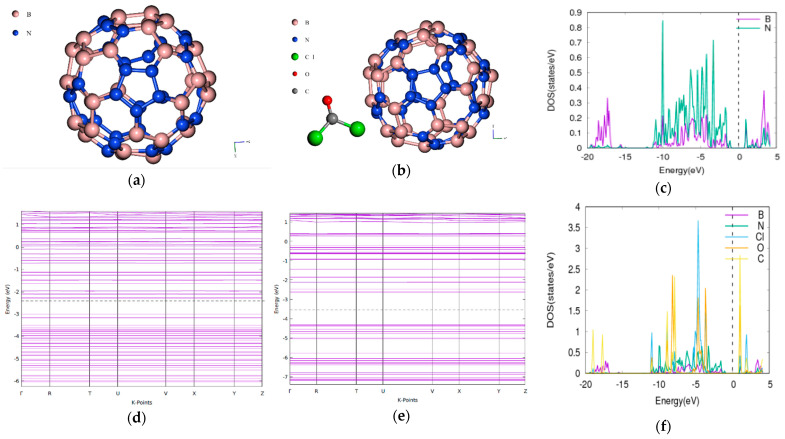
Band structure and Density of States for BN70 and Phosgene gas interaction. (**a**) BN70, (**b**) phosgene-BN70, (**c**) BN70 DOS, (**d**) BN70 band structure Eg = 0.7274 eV, (**e**) phosgene-BN70 Eg = 1.7031 eV, (**f**) phosgene-BN70 DOS.

The bandgap of the pristine BN70 is 0.7274 eV. When the phosgene was adsorbed, the combined band gap increased to 1.7031 eV indicating an increase of 134% as shown in Figure 6. The energy difference between the composite of BN70 and phosgene was found to be −1.9345 Ry.

All the calculated bands show a reasonable agreement with data already available from previous studies both experimental and Ab initio methods as indicated in Table 1.

The LDA functional is however known to underestimate the bandgap [39,40]. The differences in the adsorption energies and the various modulations in bandgap may be attributed to the different types of adsorption which can be either chemisorption or physisorption as shown in Table 2. CNT and BNNT have significant modulations to the bandgap and is corroborated by the projected density of state (PDOS) plots. For the phosgene-CNT interaction, the Cl-d and Cl-s orbitals of chlorine atoms of the phosgene were strongly adsorbed unto the carbon nanotube and contributed to altering the bandgap (Figure 1c). The phosgene-BNNT interaction had C-p and Cl-d contributing to the bandgap modulation as shown in Figure 2c. Similarly, for BN70 the Cl and C of the phosgene molecule contributed to the observed modulation of the band after the interaction (Figure 6f). Chemisorption will incorporate species onto the surface of the material, thereby altering its makeup and physical properties. However, the process of physisorption only, with no subsequent chemisorption, would only result in weak intermolecular interactions between the surface and the phosgene gas being adsorbed. These interactions do not involve formal sharing of electrons, therefore their influence on the material structure will be negligible as well their influence on the bandgap.

PBNNT, PC60, and PC70 had small decreases in the bandgap. As shown in Table 2, phosgene-BN70 had the largest increase of 134.12% with PC60 recording the largest decrease of 1.77%. The adsorption energies shown in Table 3 show the nanotubes CNT and BNNT phosgene interaction required 186,660.95 kJ/mol and 179,921.26 kJ/mol, respectively. The adsorption energies calculated show that strong covalent bond and formal sharing of electrons occurred for the phosgene-(CNT and BNNT) interaction whereas weak van der Waals forces were involved in the phosgene-BN70 interaction and the other interacting species. The huge change in the bandgap of BN70 is however interesting and further investigation to determine the mechanism of the modulation could find applications in band gap tuning.

## 4. Summary and Conclusions

This study investigated the suitability of 40 atoms, [5,0] boron nitride nanotube (BNNT), 40 atoms, [5,0] carbon nanotube (CNT), fullerene balls C60 and C70, as well as fullerene-like boron nitride nanocages, BN60 and BN70 for phosgene gas sensor device application. Density functional theory using the local density approximation to describe the exchange-correlation of the many-electron interaction was employed as opposed to the previous studies which used the sensing of phosgene gas with higher-level functionals in treatment. The higher level functionals of Perdew, Burke, and Ernzerhof (GGA-PBE) and the hybrid B3LYP-D3/def2-SVp gives more accurate results. However, the choice of LDA is satisfactory for establishing baseline suitability for determining or screening materials for sensor device fabrication. Given that the screening criteria are modulation of the conductivity on the adsorption of the gas, it can be said that pristine CNT and BNNT are unsuitable for sensor device fabrication due to the unfavourable thermodynamic energy barrier. The fullerene or nanocage materials, C60, C70, BN60, and BN70 have small but appreciable modulation of conductivity given the changes observed in the bandgap. However, C60 and C70 had decreases in their bandgap whereas the others recorded an increase in their band gaps after adsorption. In absolute terms for the magnitude of change in the bandgap, that is without regard for a decrease or increase, the following order of change is established as follows: PBN60 (0.19%) < PC70 (1.39%) < PC60 (1.77%) < PBNNT (27.64%) < PCNT (65.29%) < PBN70 (134.12%). However, it may be desirable to have increase conductivity (decrease in bandgap) after adsorption for electronic circuit design because it will mean a lower energy consumption. Thus, the decrease in bandgap and available free energy becomes the final criteria for selecting the optimal nanomaterial. Thus, C60 is most suitable as it has the largest decrease in the bandgap of 1.77%.

## Figures and Tables

**Table 1:** Summary of band gaps calculated compared with experimentally available data and other similar ab initio methods.

Model	Calculated Band Gap eV	Experimentally Determined Band Gap eV	Ab Initio Methods	References
[5,0] CNT	1.90	-	2.03 (DFT) 2.32 (TB)	[31]
[5,0] BNNT	3.41	5.5	2.73–4.6 (DFT)	[20,21,36,37]
C60	1.63	1.86 ± 0.1 (Microwave Absorption Method) 1.8 (EELS)	1.79–2.76	[32,34,38]
C70	1.72	1.57 ± 0.1(Microwave Absorption Method)	1.74–2.69	[32,38]
BN60	2.15	-	1.48–2.55	[35]
BN70	0.73	-	-	

**Table 2:** Changes in bandgap after phosgene-adsorbent interaction. Changes in bandgap after phosgene-adsorbent interaction. E_f_ is the Fermi energy and the corresponding percentage change (%Δ) in the bandgap after adsorption.

Model	E_f_ (eV)	HOMO (eV)	LUMO (eV)	E_g_ (eV)	%Δ
CNT	−0.2695	–1.2174	0.6785	1.8959	-
PCNT	0.0756	–1.4913	1.6424	3.1337	65.29↑
BNNT	0.5794	–1.1297	2.2884	3.4181	-
PBNNT	0.1400	–2.0414	2.3213	4.3627	27.64↑
C60	–3.4496	–4.0030	–2.3672	1.6358	-
PC60	–3.9954	–4.4322	–2.8253	1.6069	–1.77↓
C70	–2.6274	–3.8487	–2.1214	1.7273	-
PC70	–3.1385	–4.3108	–2.6074	1.7033	–1.39↓
BN60	–2.8907	–3.8001	–1.6547	2.1454	-
PBN60	–3.3846	–4.1290	–1.9795	2.1495	0.19↑
BN70	–2.4168	–3.0131	–2.2857	0.7274	-
PBN70	–3.6105	–4.3106	–2.6075	1.7031	134.12↑

Note: Phosgene is referred to here and subsequently with the letter P, it must not be confused with the symbol P for phosphorus. The arrows, ↑ and ↓ represent an increase or a decrease in the band gap respectively.

**Table 3:** Obtained adsorption energies of the phosgene-adsorbent nanomaterial cluster.

Model	E_ad_ (Ry)	kJ/mol
CNT	142.09	186,660.95
BNNT	136.96	179,921.26
C60	–1.93	–2539.65
C70	–1.93	–2538.68
BN60	–1.94	–2548.05
BN70	–1.93	–2541.30

## Sample Availability

Samples of the compounds are available from the authors.
